# Training-Induced Changes in Radial–Tangential Anisotropy of Visual Crowding

**DOI:** 10.1167/tvst.9.9.25

**Published:** 2020-08-17

**Authors:** Maka Malania, Maja Pawellek, Tina Plank, Mark W. Greenlee

**Affiliations:** 1 Institute for Experimental Psychology, University of Regensburg, Regensburg, Germany; 2 Children's University Hospital, University of Regensburg, Regensburg, Germany

**Keywords:** visual crowding, perceptual learning, fMRI

## Abstract

**Purpose:**

One of the diagnostic features of visual crowding, radial–tangential anisotropy, has been observed both in behavioral experiments as well as in responses of the blood-oxygenation-level-dependent (BOLD) functional magnetic resonance imaging (fMRI) signal. As has been shown previously, crowding is stronger for radially arranged flankers, and this tendency is reflected in BOLD signal suppression. In the current study, we examined the effect of practice on the neural correlates of crowding. We expected that training on a crowding task would cause shrinkage of the crowding zone that would be mirrored in corresponding BOLD signal responses.

**Methods:**

Pre- and post-training fMRI images were acquired in 17 healthy volunteers using a 3-tesla MRI scanner. Participants were trained over 4 consecutive days on a crowding task.

**Results:**

Comparison of the pre- and post-training behavioral data indicates a significant shrinkage of the crowding zone as a result of training. Moreover, we observed a pronounced radial–tangential anisotropy in the BOLD signal prior to training; that is, radial flankers induced a larger reduction in the BOLD signal compared to equally spaced tangential flankers. After training, this radial–tangential anisotropy in the BOLD signal was significantly reduced. Specifically, we found significant changes in BOLD responses for the radial flanker configuration.

**Conclusions:**

Our results demonstrate that training-induced changes in the anisotropic shape of the crowding zone are reflected in the BOLD signal in the early visual cortex.

**Translational Relevance:**

Perceptual learning tasks may have the potential to improve visual performance by promoting neural plasticity. Our results could motivate the development of suitable rehabilitation protocols for patients with central vision loss.

## Introduction

The visual environment we live in has an excessive complexity and contains multitudes of elements; therefore, our visual system often faces the challenge of identifying an object within clutter. The inability to recognize an object when it is surrounded by flanking objects is referred to as *visual crowding*.^1^^–^^3^ Crowding is considered to be the main bottleneck of object recognition in the periphery and it sets limits to visually guided actions, reading, and other tasks of everyday life. Although the neural mechanisms of crowding have been extensively discussed, many aspects of crowding remain largely unresolved.

There are several theories of crowding. One of these is masking theory. Although crowding shares some similarities with masking phenomena, it also differs in several important ways; for example, the extent of crowding depends on eccentricity whereas masking does not.^4^^,^^5^ The current belief is that crowding results from some sort of spatial pooling that occurs in peripheral vision.^2^^,^^5^ The nature of this pooling is not precisely known, and several different mechanisms have been proposed, such as averaging,^6^ population processing,^7^ or summary statistics.^8^^,^^9^ Another popular explanation for crowding is the substitution model, which implies that crowding occurs through the replacement of target features by features of the flankers.^10^^,^^11^ There is also evidence that attention can modulate the critical spacing.^12^^–^^16^ None of these models, however, can fully explain all of the characteristics of crowding.

Crowding has multiple characteristics. One of these is related to the concept of critical spacing: the minimal distance between the target and flanker that is required for accurate target recognition. Bouma^17^ proposed a linear proportionality relationship between critical target–flanker spacing and eccentricity corresponding to about half of the eccentricity of the target (Bouma's law). Another hallmark of crowding is inner/outer asymmetry, where outwardly located flankers have a stronger crowding effect than flankers located closer to the fovea.^17^^–^^21^ In addition to inner/outer asymmetry, another prominent feature of crowding is its radial–tangential anisotropy. Crowding is stronger for flankers arranged along a radial axis than along a tangential axis with respect to the fovea. The spatial extent of the crowding zone is thus elongated along the radial axis relative to the fovea.^21^^,^^22^ This anisotropy has been observed both in behavioral experiments as well as in blood-oxygenation-level-dependent (BOLD) responses measured with functional magnetic resonance imaging (fMRI).^21^^–^^26^ There is evidence that the strength and extent of crowding can be reduced by training,^27^^–^^30^ but it is still unclear how this improvement in performance is reflected in neural responses.

In the current study, we used fMRI to examine the effects of training on the neural correlates of crowding. As has been shown earlier, crowding is more pronounced for radially arranged flankers, which is reflected in the BOLD (neurophysiological) signal suppression.^31^^–^^34^ In the light of previous findings, we would expect to find changes in the BOLD signal due to practice on the gap-detection task with a Landolt C target flanked by equally sized rings. Specifically, training should reduce the crowding effect in perception and, as a consequence, should diminish crowding-induced suppression in the BOLD response.

## Materials and Methods

### Participants

Seventeen healthy volunteers (13 females, 4 males) between the ages of 20 and 27 years (mean age, 23.4 years) were recruited from the University of Regensburg student body. All subjects had normal or corrected-to-normal visual acuity and no history of psychiatric or neurological disorders. All participants provided written informed consent. They received course credits for participation but no financial compensation. Data from all 17 subjects were included in the final statistical analysis. The study was approved by the ethics committee of the University of Regensburg, and it was conducted according to the tenets of the Declaration of Helsinki.

### Behavioral Experiment

#### Stimuli and Apparatus

**Figure 1.**
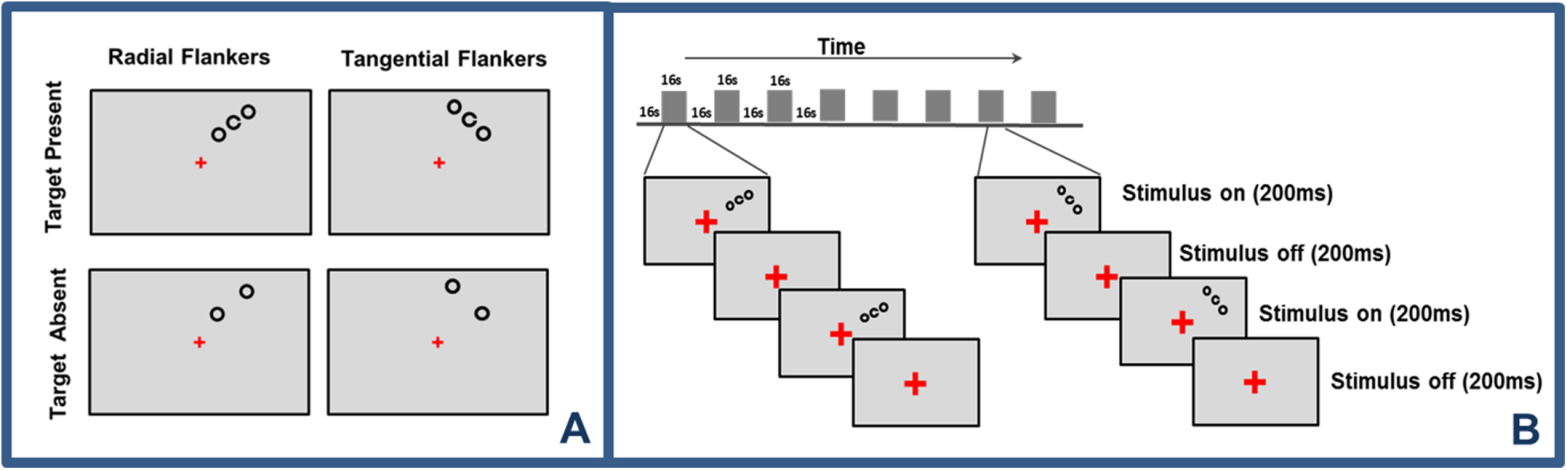
Experimental design and procedure for fMRI measurements. (**A**) Examples of target–flanker stimulus configurations. Four stimulus conditions are depicted: radial flankers with target present, radial flankers with target absent, tangential flankers with target present, and tangential flankers with target absent. (**B**) Time course of a typical fMRI run with a central fixation task. We used a block design paradigm in which each fixation block (16 seconds) was followed by a stimulation block (16 seconds). In each stimulation block, only one stimulus type was presented. The stimulus displays are not drawn to scale.

A high-contrast Landolt C optotype was used as a target stimulus that was flanked by same-sized rings with no gap (Fig. 1A). Stimuli were presented at 6.5° eccentricity in the upper-right visual field, at an angle of 25° clockwise from the vertical meridian. The stimulus size was 0.75°. Flankers were placed either radially or tangentially with respect to the fovea. The stimuli were generated and controlled by Presentation 17.0 software (Neurobehavioral Systems, Berkeley, CA). They were presented on a liquid-crystal display monitor (resolution, 1024 × 768 pixels; 75 Hz; screen size, 37.5 × 30 cm; viewing distance, 54 cm) while the participant's head rested on a chin rest. Stimuli were presented for 67 ms interleaved with a gray screen (200 ms). The luminance (gamma) function of the monitor was calibrated with a Chroma Meter CS-100 color and luminance meter (Konica Minolta, Tokyo, Japan). The background and stimulus luminance values were 151.6 cd/m^2^ and 0.2 cd/m^2^, respectively. The luminance contrast of the stimuli was defined by Weber contrast and corresponded to an absolute value of 0.998.

#### Experimental Procedure

The spatial extent of crowding was measured in psychophysical experiments with target-to-flanker distance varying randomly (ranging from 0.75° to 3° with step size of 0.5°) using the method of constant stimuli. In each trial, subjects were presented a Landolt C surrounded by two same-sized rings either radially or tangentially arranged with respect to central fixation. Subjects were required to maintain central fixation and to identify the gap direction of the Landolt C. Participants’ eye movements were monitored by a video eye tracker (Cambridge Research Systems, Rochester, UK). In a four-alternative, forced-choice task, subjects indicated their responses by pressing the left, right, up, or down arrow keys on the computer keyboard according to the Landolt C gap orientation. Psychophysical thresholds were defined for each flanker condition separately as 68% identification accuracy by fitting a psychometric function to data using a cumulative normal function (Palamedes toolbox^35^). Subjects were trained on the crowding task over 4 consecutive days (1348 trials for each flanker condition). The experiment consisted of two training blocks (about 20 to 25 minutes for each block) per day, with either radially or tangentially arranged flankers presented within a given block. The experiment was conducted in a dimly illuminated room. Subjects were given a short practice session to become familiar with the task.

### fMRI Measurements

Two MRI measurement sessions were conducted. One measurement was performed before the above-described behavioral training and the other one after training within 1 week.

#### Stimuli

As in the psychophysical experiment, we used Landolt C target stimuli and same-sized rings as flankers. Stimuli were presented at 6.5° eccentricity in the upper right visual field. Stimulus size was 0.75°. Differing from psychophysical experiments, in this fMRI experiment we used a fixed target-to-flanker distance of 0.95°. This flanker distance was selected to ensure a prominent crowding effect in all participants based on preliminary observations. Participants viewed the stimuli through a mirror fixed on the head coil. Stimuli were back projected onto a translucent screen with a calibrated PROPixx projector (VPixx Technologies, Saint-Bruno-de-Montarville, Canada). The projector was located outside of the MR scanner room, and the image was projected with a customized lens through a waveguide. Screen parameters were as follows: 1024 × 768 pixels, 60 Hz, 40 × 30 cm (24° × 18°), 95-cm viewing distance. The background and stimulus luminance values were calibrated with the same Minolta colorimeter used for the behavioral experiments and were 171.5 cd/m^2^ and 0.19 cd/m^2^, respectively. The luminance contrast of the stimuli was defined by Weber contrast corresponding to the absolute value of 0.999.

#### Experimental Procedure

During each fMRI scan, subjects maintained fixation and performed a central fixation task while the crowding stimuli were presented in the periphery. The color of the fixation cross altered from red to either green or blue on average every 2 seconds in a random manner. The subjects’ task was to indicate the color change of the fixation cross by pressing one of two buttons. Our choice of a central fixation task paradigm in the fMRI experiments was determined by the importance of maintaining stable fixation, as we measured BOLD signal changes within a small eccentric region of interest (ROI). There is evidence that the BOLD signal is reduced with increasing crowding regardless of whether attention is directed toward or away from the stimuli.^34^ There were four stimulus conditions: radially or tangentially arranged flankers with the target either present or absent (target-present and target-absent conditions). These conditions were used to tease apart the modulation in brain activity in response to the stimuli and crowding. The stimulation paradigm was adopted from an earlier study by Kwon et al.^25^ The stimuli and experimental procedure are shown in Figure 1.

We used a block design with alternating stimulation and fixation blocks of 16-second duration. Altogether, there were eight stimulation and nine fixation blocks during one run. Each block consisted of eight trials lasting 2 seconds each. In each stimulation block, only one flanker–target condition was presented. Eight fMRI runs were acquired from each subject.

Additionally, we conducted on the same day a separate scan session to define the ROIs in early visual cortex. In this scanning session, the target stimulus was presented together with radial and tangential flankers. The positions of the target and fixation point were identical to those used in the main experiment. We defined ROIs based on this independent localizer session as clusters of active voxels within the corresponding retinotopic region.

#### MRI Data Acquisition

Data were collected with a Prisma 3T scanner (Siemens, Erlangen, Germany), using a 20-channel head coil. For functional scans, an echo planar imaging sequence was used (repetition time [TR] = 2000 ms, echo time [TE] = 30 ms, flip angle = 90°, 37 slices, voxel size = 3 × 3 × 3.3 mm). For anatomical images, a high-resolution, T_1_-weighted anatomical image was acquired in the same session using a magnetization-prepared rapid acquisition with gradient echo sequence. Flip angle was 9°, TR was 2300 ms, and TE was 2.98 ms. We acquired 176 slices with 1 × 1 × 1-mm isotropic voxels.

#### Data Analyses

The T_1_-weighted structural images were processed for cortical reconstruction and segmentation using FreeSurfer version 5.3 (Martinos Center for Biomedical Imaging, Charlestown, MA) as described elsewhere.^36^ Several steps were executed. First we performed motion correction and non-brain-tissue removal.^37^ The images were then corrected for intensity nonuniformities and transformed into Talairach space.^38^^,^^39^ After segmentation of the subcortical white matter and deep gray matter, volumetric structures of the cortical surface were reconstructed based on gyral and sulcal structure.^40^^,^^41^ The reconstructed datasets were visually inspected for accuracy at several points along the processing pipeline, and segmentation errors were manually corrected, reprocessed, and reinspected.

The functional data were preprocessed using the FsFast (FreeSurfer Functional Analysis Stream) tool, which is part of the FreeSurfer software package. Briefly, processing steps included head motion correction by registering all volumes on a reference volume via rigid-body transformation, intensity normalization, spatial smoothing (kernel size of 5 mm), and co-registration of functional on structural space. Functional images were analyzed using a general linear model with an assumed hemodynamic response function.

#### Labeling ROIs

**Figure 2.**
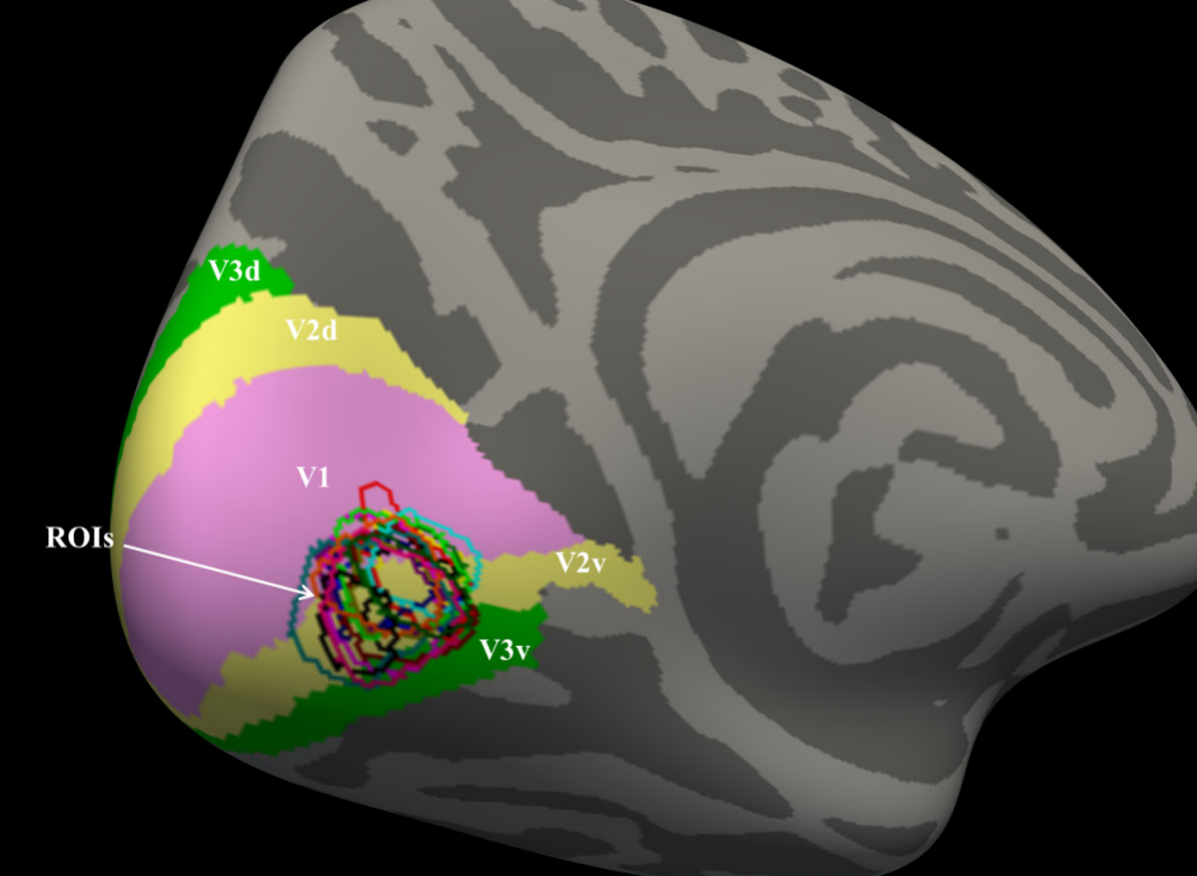
ROI definition. The ROIs of all 17 subjects (each subject's ROI is indicated by a different color) are mapped on the surface of the left hemisphere of fsAverage (MNI305) brain and retinotopic maps (V1, V2v, V2d, V3v, V3d) defined by Glasser's atlas.^42^

An ROI was defined as an area of the cortex that contained voxel clusters that responded to our stimulus during ROI localizer scan sessions. Figure 2 illustrates the retinotopic maps (V1, V2v, V2d, V3v, and V3d, denoted by different surface colors in the figure) defined by Glasser's atlas^42^ and the ROIs of all 17 subjects overlaid on the left hemisphere of the fsAverage brain surface. Statistical parametric maps were calculated based on the general linear model. The stimulation blocks were convolved using a cumulative gamma function with δ = 2.25, τ = 1.25, and α = 2 as predictors. We contrasted the stimulation (target plus four flankers, both radial and tangential) with the fixation condition. In both conditions, subjects performed the same color-change detection task. Statistical significance maps for each condition were overlaid on the inflated cortical surface of each hemisphere to visualize the BOLD activity, and ROIs were defined manually. Significance maps were adjusted to *P* ≤ 0.001. We examined the BOLD signal changes within ROIs only in the left hemisphere, as stimuli were presented in the upper right visual field.

All statistical analyses were performed using SPSS Statistics 25 (IBM Corp., Armonk, NY). Significant differences were accepted at *P* < 0.05. All error values in the figures refer to the standard error of the mean (SEM). Pre- and post-training measurements were compared using the paired-sample, *t*-test and one-way, repeated-measures analysis of variance (ANOVA). We performed a two (flanker configuration, radial or tangential) × two (target present or target absent) × two (pre-training or post-training) repeated-measures ANOVA on mean BOLD data. The critical spacing was calculated for each participant and condition separately, and the results were subjected to a two (flanker configuration, radial or tangential) × four (training day 1, day 2, day 3, or day 4) repeated-measures ANOVA.

The pre- and post-training behavioral crowding index (C_psy_) corresponds to the critical space, also known as the crowding zone (in degrees of arc), determined on the first and the last days of training.

The BOLD crowding index (C_BOLD_) is defined as the BOLD response R (percent signal change) difference between target-present (T_p_) and target-absent (T_a_) conditions, calculated for radial and tangential flankers separately.

(1) $$C_{\mathrm{BOLD}}=\left(R_{T_{p}}-R_{T_{a}}\right)$$

Additionally, the anisotropy index was calculated for behavioral (A_psy_) and for fMRI data (A_BOLD_). The anisotropy index for behavioral data is defined as the difference between radial (C_r_) and tangential (C_t_) crowding zones divided by their sum.

$$A_{\mathrm{psy}}=\left(C_{r}-C_{t}\right)/\left(C_{r}+C_{t}\right)$$

The BOLD anisotropy index is calculated as the difference between the radial and tangential BOLD crowding indices (C_BOLD-rad_ and C_BOLD-tang_, respectively) divided by the sum of BOLD responses on the radial and tangential absent conditions.

(3) $$\begin{array}{c} \;\begin{array}{c} A_{\mathrm{BOLD}}=\left[\left(R_{T_{p-\mathrm{rad}}}-R_{T_{a-\mathrm{rad}}}\right)-\left(R_{T_{p-\mathrm{tang}}}-R_{T_{a-\mathrm{tang}}}\right)\right]/\left(R_{T_{a-\mathrm{rad}}}+R_{T_{a-t\mathrm{ang}}}\right) \end{array} \end{array}$$

where R_Tp-rad_ is the BOLD response in the radial target-present condition; R_Ta-rad_ is the BOLD response in the radial target-absent condition; R_Tp-tang_ is the BOLD response in the tangential target-present condition; and R_Ta-tang_ is the BOLD response in the tangential target-absent condition. Spearman's rank correlation coefficient was calculated to evaluate possible correlations between the changes in A_psy_ and A_BOLD_. In addition, a post hoc power analysis was performed to define the appropriate sample size.

## Results

**Figure 3.**
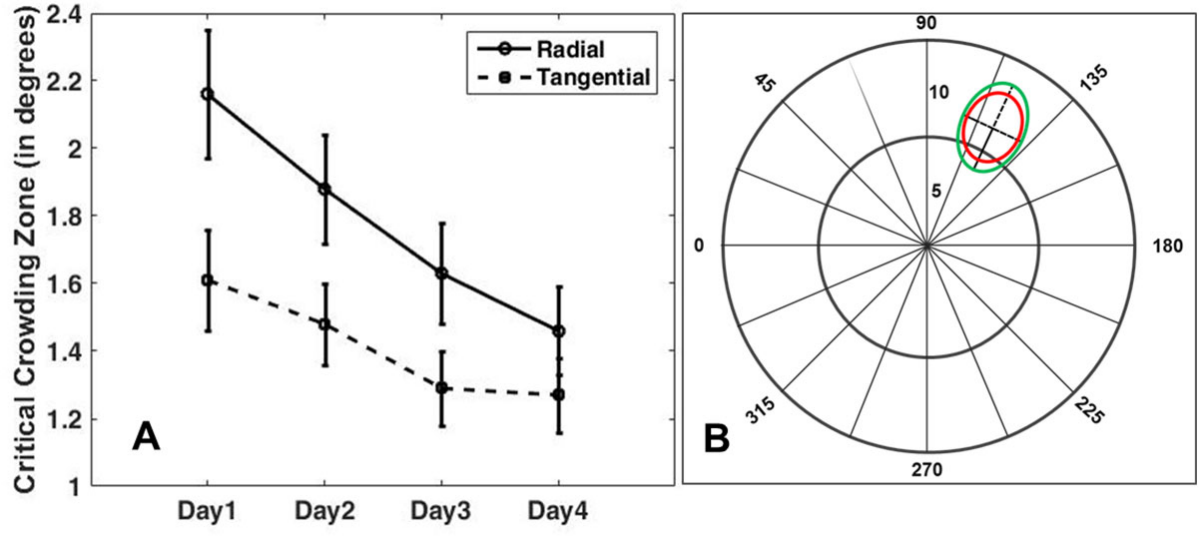
Results of behavioral training. (**A**) Day-by-day mean critical spacing thresholds are averaged across all 17 subjects. The mean pre-training critical target–flanker spacing was 2.16 ± 0.2° radially and 1.61 ± 0.15° tangentially. The post-training critical target–flanker spacing changed to 1.46 ± 0.13° radially and 1.05 ± 0.11° tangentially. Error bars denote ± 1 SEM. (**B**) The mean pre- and post-training changes of crowding zones are drawn as *green* and *red ellipsoids*. After training, the crowding zone shrank significantly in the radial axis direction.

The results of the psychophysical measurements are presented in Figure 3. A 2 × 4 factorial design ANOVA (flanker configuration and training sessions as within-subject factors) was used to assess the statistical significance of critical-space changes before and after training for two different flanker types. We observed a significant main effect of training, *F*(3, 48) = 13.2 and *P* = 0.0001, and flanker configuration, *F*(1, 16) = 12.5 and *P* = 0.003, as well as a significant interaction effect, *F*(3, 48) = 2.94 and *P* = 0.042. In addition, paired *t*-tests revealed a significant improvement in performance after training in both radial and tangential flanker conditions (*P* = 0.0001 for radial and *P* = 0.008 for tangential). The scaled pre- and post-training crowding zones are shown in Figure 3B as green and red outlined ellipsoids, respectively. There was a statistically significant (*P* = 0.03) reduction of the radial–tangential ratio from 1.42 to 1.18 as a consequence of training (i.e., after training the crowding zone became less anisotropic).

**Figure 4.**
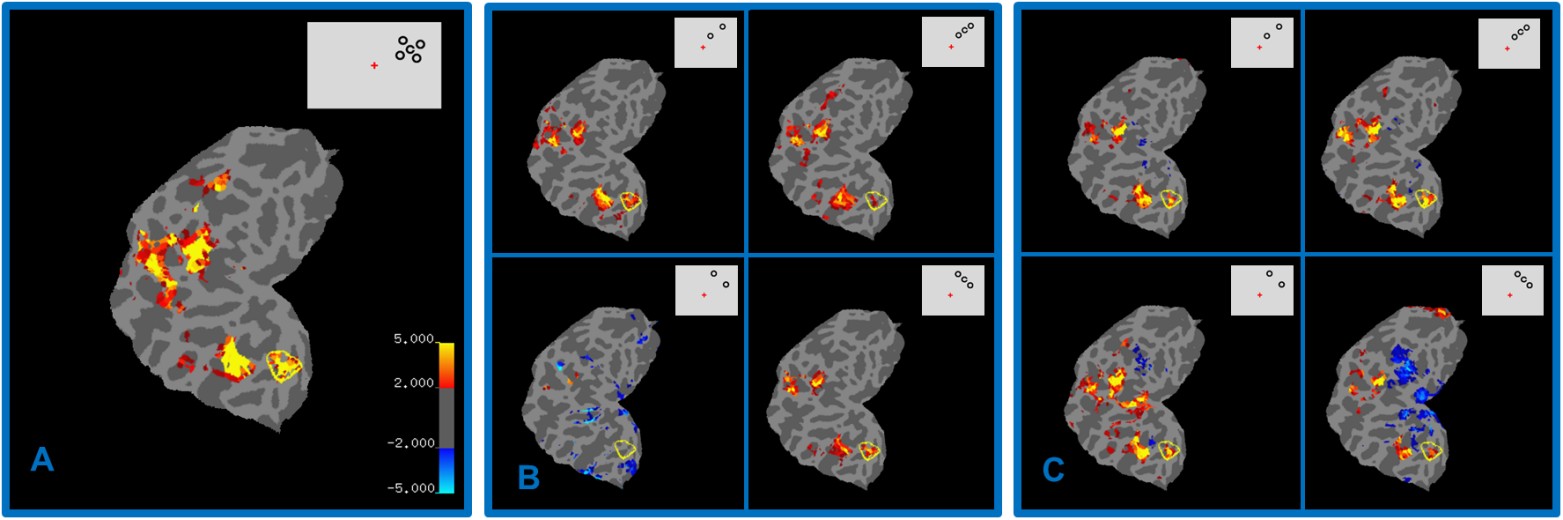
BOLD activation maps displayed on the flat occipital patch of one representative participant. Significant activations are depicted in warm colors (*red*, *orange*, and *yellow*) and deactivations in *blue*. The ROI borders are indicated by *yellow* *lines*. (**A**) Statistical parametric maps for the ROI localizer runs are shown for one representative subject. Activity maps were overlaid on the flat occipital patch that was cut along the calcarine sulcus. False positives were reduced by adjusting the significance maps by a false-discovery rate of 0.001. ROIs were defined as areas of the cortex that contained voxel clusters that responded to our stimulus during ROI localizer scan sessions. An example of the stimulus presented during a ROI localizer run is shown in the upper right corner. (**B**, **C**) Activation maps overlaid on the occipital patch for four different stimulus conditions in pre- and post-training sessions, respectively: radial flankers with target absent and radial flankers with target present (*upper row*) and tangential flankers with target absent and tangential flankers with target present (*lower row*). The left panel, **B**, corresponds to pre-training and the right panel, **C**, to post-training MRI measurements in one representative subject. The corresponding stimuli for each condition are presented on the upper right corners. The percent signal change of the BOLD signal was calculated for the voxels located within the ROIs.

The results of the fMRI measurements are presented in Figure 4 for one representative participant. The percent signal change was extracted from voxels that belong to the ROI for each stimulus condition separately. Figure 4A presents a flattened left occipital lobe of this participant. We observed a strong radial–tangential anisotropy in the BOLD signal prior to training; that is, there was a strong reduction in BOLD signal in the radial target-present condition compared to the target-absent condition, thereby replicating the results of Kwon et al.^25^ Adding a target to tangentially arranged flankers produced a stronger BOLD signal compared to the target-absent condition. This distinction in neuronal activation can also be visualized with activation maps such as those shown in Figures 4B and 4C based on the same participant.

**Figure 5.**
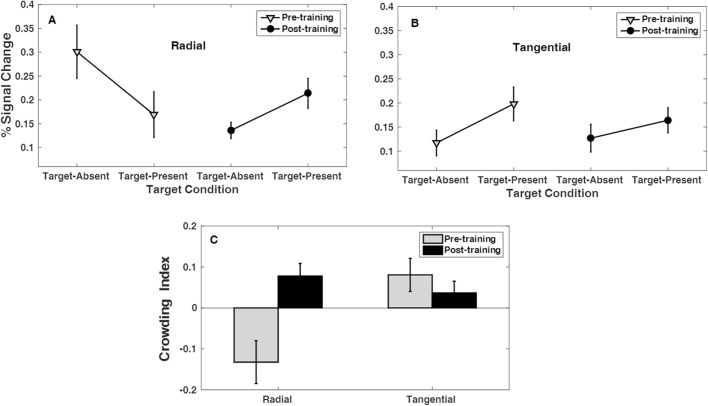
Percentage of BOLD signal change and BOLD crowding index (C_BOLD_). Shown are the results of pre- and post-training BOLD signal measurements obtained from the ROI regions for radial flanker conditions (**A**) and for tangential flanker conditions (**B**), while adding or removing central target stimuli to flankers. The figure illustrates the mean percentage of BOLD signal averaged across 17 subjects. Error bars denote ± 1 SEM. (**C**) Changes in the BOLD crowding index (C_BOLD_) in pre- and post-training sessions for tangential and radial flankers. The plotted error values are standard errors of the means. The positive values indicate a weaker crowding effect; the negative values are indicators of a stronger crowding effect.

After training, the BOLD signal increased in the target-present radial flanker condition compared to the target-absent condition; that is, we observed a pattern of BOLD activity that was reversed compared to that found in the pretraining session. Figures 5A and 5B illustrates the mean percent signal changes of BOLD responses averaged across the 17 subjects in pre- and post-training measurements for tangential and radial flanker configurations separately. A repeated-measures ANOVA of the BOLD signal for all four conditions in pre- and post-training sessions indicated a significant main effect on flanker configuration, radial versus tangential *F*(1, 16) = 6.4 and *P* = 0.023, as well as a significant interaction between flanker configuration (radial or tangential) and target present or target absent conditions, which is an indicator of the anisotropy effect, *F*(1, 16) = 6.74 and *P* = 0.019. Most importantly, we observed a significant three-way interaction among flanker configuration (radial or tangential), target present or target absent, and pre-training/post-training conditions, *F*(1, 16) = 7.4 and *P* = 0.015, which is an indicator of a learning effect on BOLD signal anisotropy.

To examine this learning effect on a specific flanker configuration, we performed a paired-sample *t*-test on the pre- and post-training BOLD crowding index C_BOLD_ (Equation 1). We suggest that comparison of crowding indices gives more relevant measures of the BOLD signal changes due to training than the comparison of absolute BOLD signal values. Values for the BOLD crowding index (C_BOLD_) are plotted as a bar plot (Fig. 5C) The results of statistical analyses showed significant changes of the BOLD crowding index (C_BOLD_) for the radial flanker configuration (*P* = 0.008) as a result of training, whereas no significant change for the tangential flanker configuration (*P* = 0.45) was evident. The repeated-measures ANOVA for the BOLD crowding index (C_BOLD_) revealed a significant main effect of flanker configuration, *F*(1, 16) = 6.7 and *P* = 0.021, and a significant interaction between flanker configuration and training, *F*(1, 16) = 7.42 and *P* = 0.015, but no significant changes were found for the main effect of training (*P* = 0.07).

**Figure 6.**
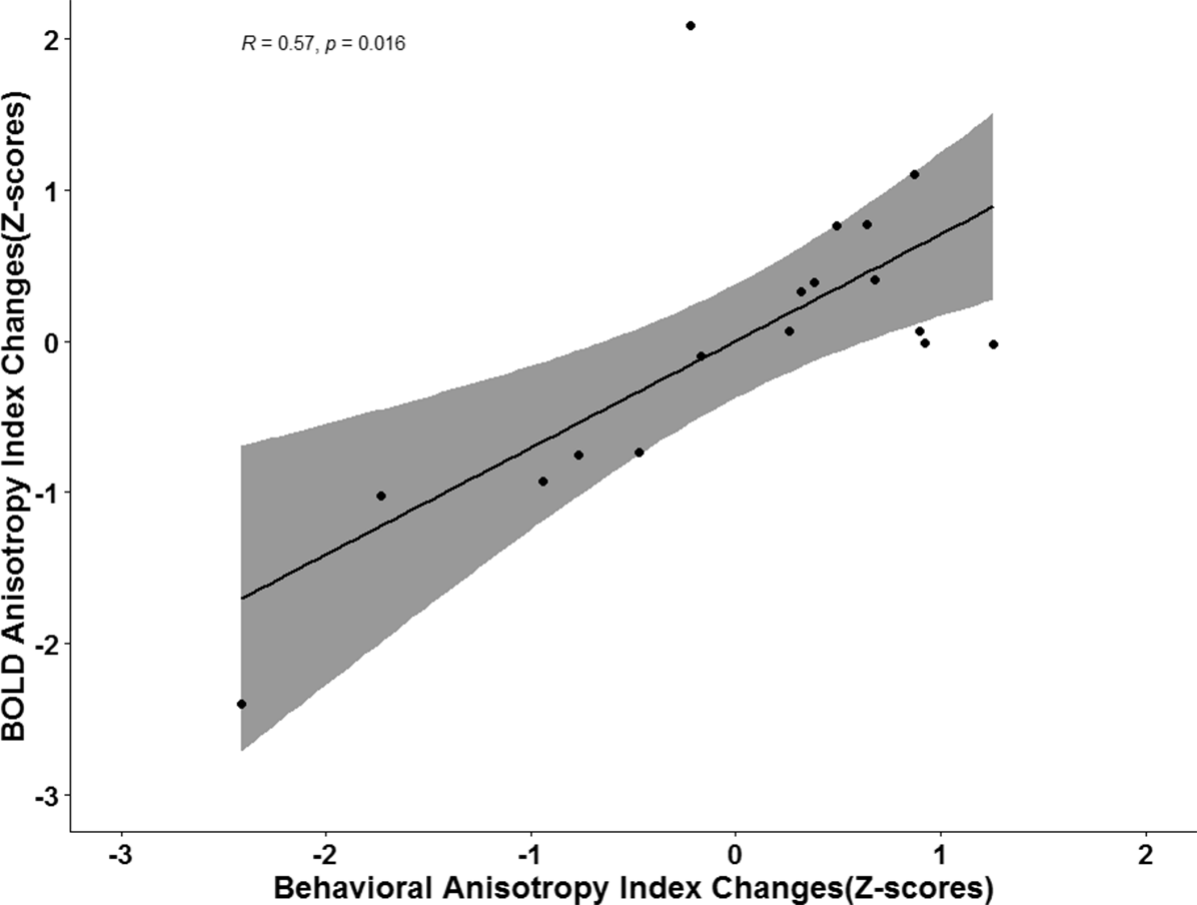
Correlation between the standardized training-induced changes in A_BOLD_ and A_psy_. We found significant positive correlation between behavioral and BOLD anisotropy index changes. The regression line (*dark black line*) is fitted to the data of all 17 subjects. *Gray shadings* represent 95% confidence intervals.

Paired-sample *t*-tests indicated significant changes in both behavioral and BOLD anisotropy indices (*P* = 0.035 for behavioral crowding index and *P* = 0.01 for BOLD signal changes) between pre- and post-training. A Spearman's rank correlation coefficient was computed to assess the relationship between the training-induced changes in A_BOLD_ and A_psy_. The scatterplots between the respective anisotropy indices are presented in Figure 6. There is a significant positive correlation between the behavioral anisotropy index and BOLD anisotropy index changes (*n* = 17, *R_s_* = 0.57, *P* = 0.016).

## Discussion

The goal of the present study was to investigate whether it is possible to reduce the effects of visual crowding through training and how such a reduction is mirrored at the level of neural responses, particularly in the BOLD signal in early visual cortex. Determining whether or not there is a substantial reduction in crowding after training has important implications for clinical practice, as well as for our understanding of peripheral vision and object recognition. Here we argue that training can modify the anisotropic feature of crowding manifested not only in behavioral responses but also at the level of neural signal changes.

Performance on different tasks is known to improve via practice (i.e., perceptual learning). This improvement usually occurs fairly quickly and enhances performance on that task even for day or several weeks or months after cessation of training.^43^^,^^44^ It is suggested that the improvement in visual perceptual functions is achieved by promoting neural plasticity as early as in the striate^45^^–^^48^ and/or in extrastriate^49^ visual cortex.

Training-induced reductions in the strength and extent of crowding have been demonstrated previously in the periphery of normal-sighted subjects,^27^^,^^29^^,^^50^^,^^51^ as well as in amblyopic individuals.^28^ Chung^24^ found a reduction in the radial–tangential anisotropy in the zone of the preferred retinal locus (PRL) in patients with age-related macular degeneration. The change in the shape of the crowding zone at the PRL was associated with more intense usage of the PRL. Similarly, after training, we observed shrinking of the crowding zone along the radial axis in our behavioral experiments, as the shape of the crowding zone became less elliptical (Fig. 3B). This indicates that the radial–tangential anisotropy of the crowding zone can indeed be altered by training.

Several studies have shown that the anisotropic feature of visual crowding is replicated in BOLD responses.^25^^,^^31^ Likewise, in our pre-training study, we observed anisotropy in BOLD responses, as adding a target to equally spaced radial flankers induced a larger reduction in the BOLD signal compared to tangential flankers. After behavioral training, the BOLD crowding index (C_BOLD_) was reduced significantly for the radial flanker condition but did not change for the tangential flanker configuration. The results, presented in Figure 5C, demonstrate that training led to a radial-specific reduction of the crowding effect (positive values of C_BOLD_ indicate a weaker crowding effect, whereas negative values indicate a stronger crowding effect). The results of ANOVA analysis for C_BOLD_ based on the fMRI BOLD response point to a significant interaction between flanker configuration (radial vs. tangential) and training effect. Also, paired-sample *t*-tests revealed significant differences in the pre- and post-training anisotropy index (A_BOLD_). Spearman's rank correlation coefficient test revealed a significant correlation between the standardized training-induced changes in A_BOLD_ and A_psy_, indicating that the greater the training-induced change in A_psy_ the larger the changes in A_BOLD_ across participants_._ To the best of our knowledge, our study demonstrates for the first time that training-induced changes in the anisotropic shape of the crowding zone are also reflected at the level of the BOLD signal in early visual cortex.

An interesting tendency in the BOLD signal changes that we observed was an increase of BOLD signal in the post-training radial–target-present condition compared to pre-training measurements, whereas the radial–target-absent condition showed the opposite trend. Three effects could contribute to the results of Figures 5A and 5B: (1) radial versus tangential bias in the fMRI signal strength, which was also reported in previous studies by Sasaki et al.^52^ and Kwon et al.^25^; (2) the repetition suppression phenomena,^53^^,^^54^ where the neural responses to repeated stimuli are reduced relative to novel stimuli when stimuli are presented multiple times; and (3) BOLD response suppression due to crowding in the pre-training radial–target-present condition and an increase in BOLD signal intensity in the post-training radial–target-present condition. This last effect presumably is induced by the training effect that leads to weakening crowding-related BOLD signal suppression. We also noticed an unexpectedly strong reduction of BOLD signal in the post-training radial–target-absent condition compared to the pre-training radial–target-absent condition, for which we currently do not have an explanation.

Multiple models attempted to explain the asymmetry of the anisotropy features of crowding. Dayan and Solomon^55^ proposed a quantitative model to interpret several paradoxical properties of crowding. In particular, their model tries to describe the inward–outward asymmetry of crowding by optimal (Bayesian) inference operating over spatially extended receptive fields. Another approach, developed by van den Berg and colleagues,^26^ is based on the principles of population coding. Those models predict several properties of crowding but still cannot explain the radial–tangential anisotropy.

An alternative explanation for the elliptical shape of the crowding zone was proposed by Nandy and Tjan,^56^ who implicated physiological and anatomical properties specific to V1 and saccade-confounded image statistics. They have suggested that normal saccadic eye movements that are radial with respect to the fovea will lead to the acquisition of inaccurate image statistics in peripheral vision. However, this theory does not predict whether crowding will lead to a decrease or an increase in neural activity.

What might be the possible neural mechanisms of training-induced changes in the anisotropic feature of crowding that we observed in our experiments? Allegedly, perceptual learning affects almost every level of processing in the brain, from synaptic connections to global patterns of blood flow.^43^ However, the neural mechanisms underlying the training effect remain highly controversial. Based on the suggestion that there is a link between BOLD signal and synaptically generated local field potentials,^57^^–^^59^ one can hypothesize that an increase of BOLD signal after training might be induced by an increase in the number or strength of synaptic connections. A new study by Contemori et al.^60^ investigating the effect of transcranial random noise stimulation (tRNS) on perceptual learning demonstrated a stronger reduction of crowding in the tRNS group compared to the sham stimulation group. They proposed that the boosting effect of tRNS could be evoked from a general increase in cortical excitability, as well as by an improved signal-to-noise ratio. Earlier studies on monkeys have shown a so-called push–pull response pattern in V1 neurons over the course of training on a contour detection task, such that responses of neurons with receptive fields lying on the contour are progressively enhanced, and those on the noisy background are suppressed.^61^^,^^62^

More recently, based on fMRI data, it has been proposed that visual perceptual learning could be associated with the two types of plasticity processes, feature based or task based.^63^ Feature-based plasticity implies that there are changes in tuning properties of the neuronal representations of a trained visual feature.^45^^,^^64^^–^^66^ Task-based plasticity refers to improvement in task-related processing due to training on a task and is characterized by connectivity changes between feature representations and decision units.^67^^,^^68^ Neural correlates of feature-based plasticity can be observed during passive viewing condition, when subjects are passively exposed to the trained stimuli. Task-based plasticity, on the other hand, is assessed during active-task conditions, when subjects are actively performing the task.

Given all of these considerations, we suggest that reduced crowding after training can be associated with an enhanced signal-to-noise ratio of neural responses to the stimuli by refinement of neural population codes in early visual cortex that represent the trained stimulus features. The reduction of the anisotropy effect in the radial direction could be explained by the presence of different lateral interactions in the periphery that exhibit training-dependent modulation.^69^ This suggestion is supported by an earlier fMRI study on orientation discrimination, the results of which indicate that training enhances the discriminability of individual neural populations (represented by individual voxels) without altering the overall activation strength, indicating that the learning-induced modulatory effects could be facilitatory for some neural populations but inhibitory for the others in response to the trained stimulus.^70^

Another interesting observation we made in our study is related to the loci of the BOLD activation during crowding. We found that most ROIs fall between visual areas V1 and V2 (Fig. 2). There are debates regarding the loci where crowding first emerges. Whitney and Levi^71^ promoted a multiple-level hypothesis of visual processing occurring as early as V1. Kwon et al.^25^ showed that anisotropy in BOLD response occurs as early as V1. On the other hand, there are studies showing evidence for learning in visual areas V2^9^ and V4,^72^ as well as high-level visual areas such as the lateral occipital cortex,^33^ and these could be the candidate loci for visual crowding. In our study, we did not perform individual retinotopic mapping; therefore, it is not possible to precisely define the exact borders between early visual areas involved in crowding.

In summary, the results of our study indicate that training reduces visual crowding and its anisotropy feature that is reflected in BOLD responses in early visual cortex. Our results support the effectiveness of plasticity-based approaches to improve visual function. We suggest that further studies, similar to those that have investigated the size and shape of population receptive fields, are required to reveal the processes that underlie training effects on crowding.
